# Draft Genome Sequences of Two Strains of Enterococcus lactis Showing High Potential as Cattle Probiotic Supplements

**DOI:** 10.1128/MRA.00436-21

**Published:** 2021-12-09

**Authors:** A. A. Korzhenkov, K. O. Petrova, A. O. Izotova, T. E. Shustikova, A. A. Kanikovskaya, E. V. Patrusheva, M. V. Patrushev, S. V. Toshchakov

**Affiliations:** a NRC Kurchatov Institute, Moscow, Russia; b NRC Kurchatov Institute-GosNIIgenetika, Moscow, Russia; Loyola University Chicago

## Abstract

Probiotic supplements are currently widely used in cattle feeding practices. However, knowledge regarding the genomic landscape of cattle probiotic microorganisms is relatively scarce and is based on analogies with human probiotics. Here, we report on the draft genome sequences of two Enterococcus lactis strains, VKPM B-4989 and VKPM B-4992, which were isolated from the rumen of a healthy calf and utilized as a probiotic additive.

## ANNOUNCEMENT

Probiotic additives have been shown to improve productivity and fitness characteristics of cattle and to reduce methane emissions from farms and agricultural companies (1). Lactic acid bacteria, primarily belonging to the genera *Bifidobacterium*, *Enterococcus*, and *Lactococcus*, represent the predominant class of microbial additives, reported to improve weight gain and feed efficiency (2, 3). Widespread representatives of the genus *Enterococcus* are currently widely utilized as a basis for human food supplements (4) and were recently shown to demonstrate significant potential as cattle probiotic additives, having a positive effect on the diversity of rumen microbiota (5). Here, we report the draft genome sequences of two Enterococcus lactis strains, VKPM B-4989 and VKPM B-4992, which were isolated from the rumen of a healthy calf in 1989 in STC Lekbiotech (Moscow, USSR). Later the strains were used as a probiotic additive (6, 7).

Before DNA isolation, the strains were cultured in MRS medium for 36 h at 28°C to 30°C under aerobic conditions. The genomic DNA was extracted as described previously (8). Fragment genomic libraries were prepared with a Nextera XT reagent kit (Illumina, San Diego, CA, USA) according to the manufacturer’s recommendations. Sequencing was performed on an Illumina MiSeq system using a 2 × 250-bp MiSeq reagent kit.

Quality control of the reads was performed using fastp v0.20.1 (9). Read processing and genome assembly were conducted with the ZGA pipeline v0.0.9 (10). Reads were trimmed based on quality (qtrim=r, trimq=18), and Illumina adapter sequences were removed with BBduk v38.75; overlapping paired reads were merged with BBmerge v38.75 (11). The remaining paired reads and merged reads were assembled with the SPAdes v3.14.0 assembler (12) using the --careful option. Assembled genomes were annotated using the NCBI Prokaryotic Genome Annotation Pipeline v5.1 (13). Unless stated otherwise, default parameters were used for all software tools.

Genome-based taxonomic classification of the sequenced strains was performed with the Type Strain Genome Server (TYGS) (14). TYGS analysis identified strains B-4989 and B-4992 as E. lactis with high confidence (digital DNA-DNA hybridization [dDDH] values of >90%) (Table 1).

**TABLE 1:** Genome sequencing of E. lactis strains

Strain	No. of raw sequencing reads	Genome size (bp)	No. of contigs	*N*_50_ (bp)	Mean genome coverage (×)	G+C content (%)	GenBank accession no.
VKPM B-4899	509,264	2,831,734	77	140,067	70	38.19	JAGPYO000000000.1
VKPM B-4992	580,275	2,643,596	66	97,582	93	38.27	JAGPYP000000000.1

Antibiotic resistance analysis performed with the Comprehensive Antibiotic Resistance Database (CARD) Resistance Gene Identifier (RGI) web server v5.2.0 (15), using Perfect and Strict hits only search criteria, discovered two antibiotic resistance genes. The identified genes were present in both strains; AAC(6′)-Ib homologs are involved in aminoglycoside antibiotic inactivation, and ABC-F ribosomal protection protein homologs are involved in antibiotic resistance via ribosomal protection.

A search of secondary metabolite gene clusters was carried out with antiSMASH (16). A type III polyketide synthase (PKS) gene cluster was found in both genomes. The genome of strain B-4989 includes two ribosomally synthesized and posttranslationally modified peptide product (RiPP)-like gene clusters, both of which encode class II bacteriocin and related biosynthetic enzymes and transporters. The genome of strain B-4992 includes two nonribosomal peptide synthetase clusters.

An NCBI search showed a lack of sequenced genomes of cattle probiotic microorganisms. E. lactis strains VKPM B-4989 and VPKM B-4992 possess antibiotic resistance genes and secondary metabolite gene clusters; however, in-depth analysis is needed to characterize cattle-specific genetic determinants of their probiotic properties.

### Data availability.

The genomes have been deposited in NCBI GenBank and are available under accession numbers JAGPYO000000000.1 (E. lactis strain B-4989) and JAGPYP000000000.1 (E. lactis strain B-4992). Raw sequencing reads are available in the NCBI SRA under accession numbers SRX10881548 (E. lactis strain B-4989) and SRX10881549 (E. lactis strain B-4992).
